# The Emerging Roles of Endocrine Hormones in Different Arthritic Disorders

**DOI:** 10.3389/fendo.2021.620920

**Published:** 2021-05-21

**Authors:** Eugenia Bertoldo, Giovanni Adami, Maurizio Rossini, Alessandro Giollo, Giovanni Orsolini, Ombretta Viapiana, Davide Gatti, Angelo Fassio

**Affiliations:** Rheumatology Unit, Department of Medicine, University of Verona, Verona, Italy

**Keywords:** bone metabolism, hormones, bone turnover markers, rheumatic disorders, parathyroid hormone

## Abstract

The relationship between endocrine hormones and the spectrum of rheumatic conditions has long been discussed in the literature, focusing primarily on sexual hormones, such as estrogens, androgens, prolactin (PRL). Estrogens are indeed involved in the pathogenesis of the main inflammatory arthritis thanks to their effects on the immune system, both stimulatory and inhibitory. The PRL system has been discovered in synovial tissue of rheumatoid arthritis (RA) and psoriatic arthritis (PsA), patients and has been propose as a new potential therapeutic target. Besides sexual hormones, in the last years scientific interest about the crosstalk of immune system with other class of hormones has grown. Hormones acting on the bone tissue (i.e. parathyroid hormone, vitamin D) and modulators of the Wnt pathway (i.e. Dickkopf-1) have been demonstrated to play active role in inflammatory arthritis course, defining a new field of research named osteoimmunology. PTH, which is one of the main determinants of Dkkopf-1, plays a crucial role in bone erosions in RA and a correlation between PTH, Trabecular Bone Score (TBS) and disease activity has been found in ankylosing spondylitis (AS). In PSA is under studying the interaction among IL-17 and bone metabolism. The purpose of this review is to discuss and summarize the recent data about the interaction between endocrine hormone and immune system in the main rheumatic disorders, covering in particular the role of bone-related hormones and cytokines. We will describe this relationship from a biochemical, diagnostic and therapeutic perspective, with a particular focus on RA, PsA and AS.

## Introduction

Hormones are involved in various aspects of the immune response and rheumatic diseases. To date, there is a considerable body of evidence on the relationship between sex hormones and autoimmunity. In recent years, the scientific interest in the crosstalk between hormones and cytokines acting on bone metabolism has also grown, even in rheumatology. Emblematic examples are arthritic disorders such as RA, PsA, and AS.

Bone tissue is nowadays considered an ‘osteo-immune’ system and a principal actor in the pathogenesis of many rheumatic diseases; for those reasons, in the last decade, the term “osteoimmunology” has been increasingly used (1, 2).

**Figure 1:**
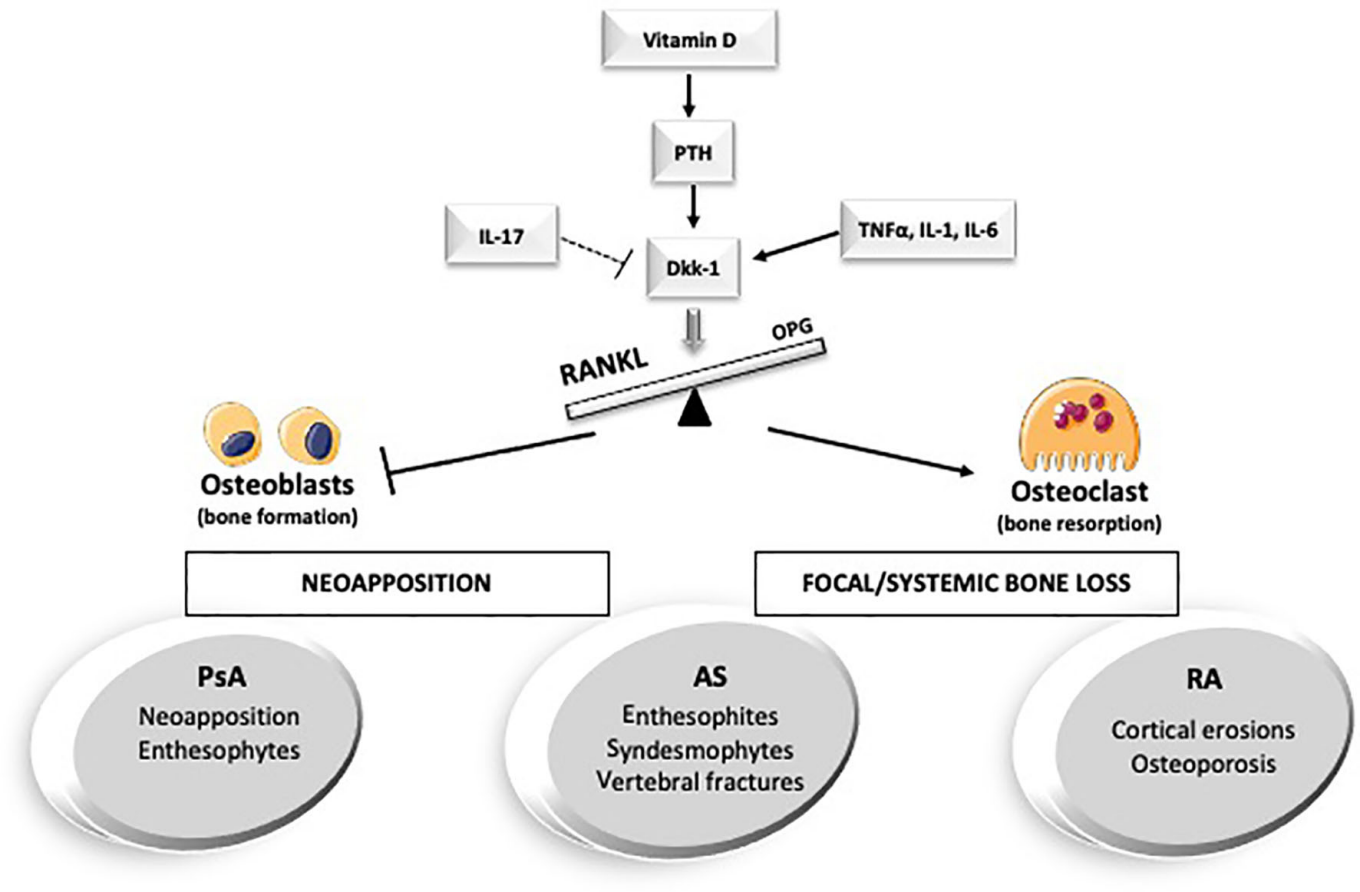
Hormones and cytokines involved in bone metabolism in the main arthritic disorders. AS, anskylosing spondilits; Dkk-1, Dickopf-1; IL-, interleukin-; PsA, Psoriatic Arthritis; PTH, parathyroid hormone; RA, Rheumatoid Arthritis; TNFalfa, Tumor necrosis factor alpha.

In the following paragraphs, we will discuss and review the main concepts and the latest findings on the interplay between hormones, cytokines, and bone in the main arthritic conditions from a biochemical, diagnostic, and therapeutic perspective without attempting to be comprehensive (**Figure 1**).

Bone remodeling is principally enacted by three types of cells: osteoblasts, osteoclasts, and osteocytes. One of the primary regulatory pathways of bone turnover is the Wnt/beta-catenin signaling (the canonical Wnt pathway) (3). Wnt signaling induces the commitment of the mesenchymal stem cell toward the osteoblast line (osteoblastogenesis) and favors their maturation and survival. In addition, in certain circumstances, it can also reduce osteoclastogenesis and bone resorption by promoting the osteoprotegerin (OPG) expression from the osteoblasts themselves (4). Dickkopf-related protein 1 (Dkk-1) is a secretory glycoprotein mainly expressed by osteoblasts and bone marrow stromal cells in the late phase of osteoblast differentiation. Dkk-1 is a potent inhibitor of the Wnt canonical pathway (5). Its role has also been investigated in various pathological conditions: low Dkk-1 and sclerostin serum levels have been described in diffuse idiopathic hyperostosis (6, 7), while its excessive overexpression seems to correlate with osteolytic lesions in multiple myeloma (8), with cortical erosions, low bone formation, and secondary osteoporosis in rheumatoid arthritis (9, 10).

Scloeristin is another inhibitor of Wnt signaling, and it is secreted mostly by osteocytes (11). Among the factors that influence Dkk-1 and sclerostin, we find several hormones, such as estrogens, androgens, parathyroid hormone (PTH) and vitamin D (12).

**Figure 2:**
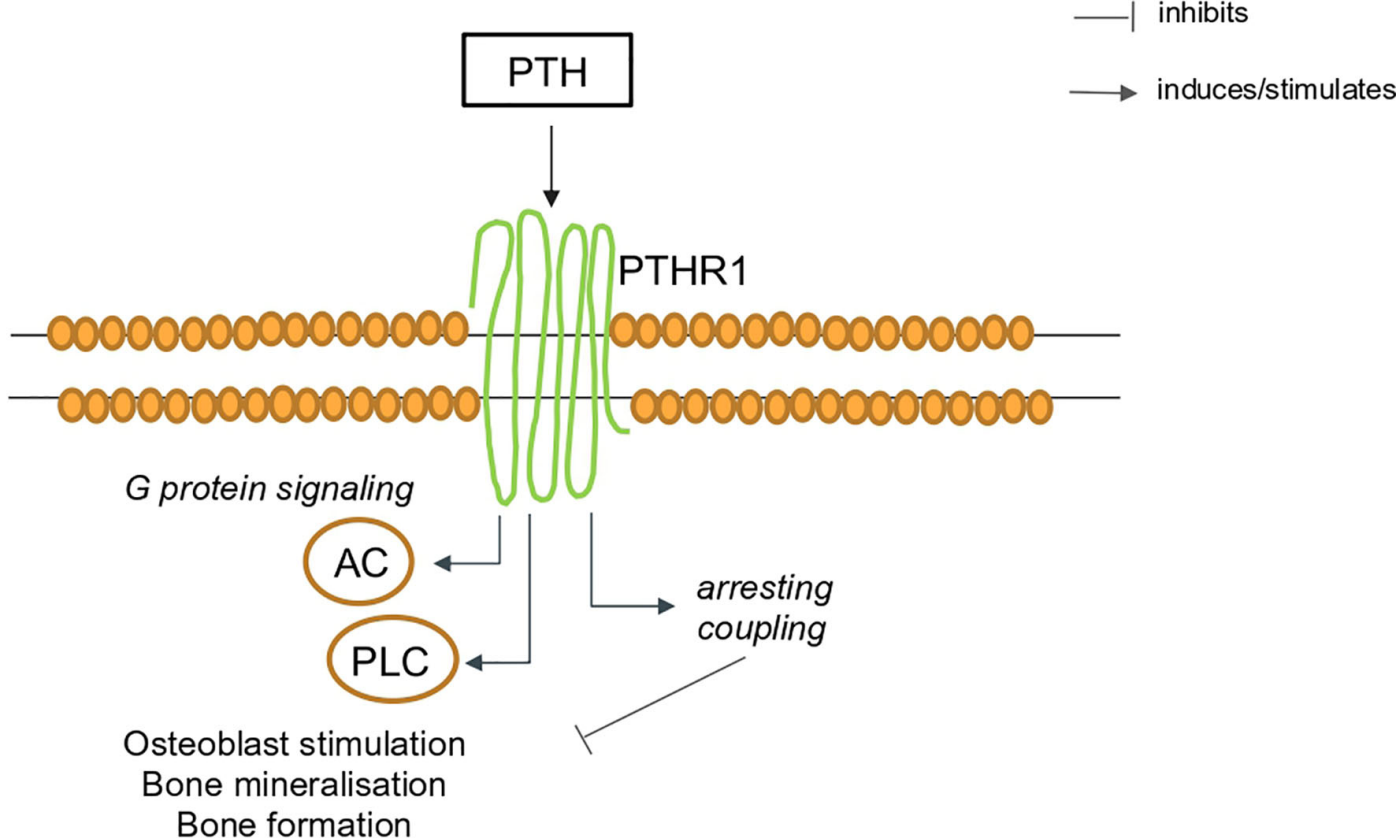
Schematic representation of PTH signaling *via* the PTH1R in bone. AC, adenylate cyclase; PTH, parathyroid hormone; PTH1R, parathyroid hormone 1 receptor, PLC, phospholipase C.

PTH is a polypeptide secreted by the parathyroid glands in response to decreases in plasma calcium, other regulators of PTH are 1,25- dihydroxyvitamin D, serum phosphate levels, and the phosphaturic hormone fibroblast growth factor-23 (FGF23). PTH acts *via* its own G protein-coupled receptors (GPCR) (**Figure 2**), a transmembrane protein expressed in different organs (13, 14). Parathyroid hormone 1 receptor (PTH1R) is expressed in bone and kidney and regulates calcium ion homeostasis through activation of adenylate cyclase and phospholipase C and the parathyroid hormone 2 receptor (PTH2R) that is expressed primarily in the central nervous system, pancreas, testis, and placenta.

Vitamin D hormone, once metabolically converted in its active metabolite, 1,25-dihydroxyvitamin D [1,25(OH)_2_D], plays an essential role in calcium homeostasis and bone metabolism. Vitamin D, by acting on different pathways, can also modulate both the innate and adaptive systems thanks to its ubiquitously distributed cellular receptor (vitamin D receptor or VDR), which alters the transcription rates of target genes responsible for the biological responses.

Another key molecule involved in bone turnover and the immune system is the receptor activator of nuclear factor-kB ligand (RANKL). Its stimulation on the macrophage/dendritic progenitors leads to osteoclast differentiation (15), and its expression has been observed not only in the bone marrow but also in lymphocytes and in the lymphoid tissues (16), where it regulates the development of immune tolerance (7).

In recent years, lymphocytes, particularly T cells, have been found to play an important role in the bone health regulation (8). Lymphocytes play a dual role in the modulation of bone remodeling: on one side, resting T cells, *via* INFγ, inhibit *in vitro* osteoclast differentiation; on the other, activated T cells partake in the osteoclasts development (17).

## Ankylosing Spondylitis

AS is a chronic rheumatic disease characterized by inflammation and extensive remodeling of the spine and joints. Eventually, it can lead to the development of spinal syndesmophytes and extra-articular enthesophytes (18). AS belongs to the spondyloarthritis (SpA) spectrum, such as PsA and axial spondyloarthritis (axSpA). These diseases are characterized by pathologic bone formation involving primarily the entheses complex and, at the same time, by cortical bone erosions (19).

Moreover, AS shows a dichotomous relationship with bone metabolism: the pathological neoformation coexists with an increased risk of fracture and impaired bone mineral density (BMD) (20, 21) (**Figure 1**).

The Wnt pathway seems to be one of the key players involved in the dual relationship with systemic bone metabolism found in AS. Unsurprisingly, the role of Dkk-1 in this disease has been the object of several investigations (22, 23). Rossini M. et al. (24) observed decreased serum levels of both Dkk-1 and sclerostin, suggesting a link between excessive Wnt exposure and the new focal bone formation. The Authors also reported a negative association between Dkk-1, spinal BMD, and vertebral fractures.

One metanalysis published in 2018 concluded that Dkk-1 serum levels in AS patients seem to be comparable to healthy controls (25). However, when the subanalysis considered only the studies characterized by high degrees of structural involvement (modified Stoke AS Spine Score >30), or increased levels of serum C reactive protein (CRP), Dkk-1 was found to be significantly reduced (25).

In addition, in AS patients, the incidence of osteoporosis is varying from 18.7 to 62%, according to studies (26). The risk of fracture is increased compared (27), despite normal or only slightly reduced BMD values (28, 29).

Boussoualim K et al. evaluated AS patients through the measurement of the Trabecular Bone Score (TBS) (30), an algorithm that leads a better assessment of vertebral bone quality (31), and documented an association between lower TBS values and disease activity and, an inverse association between TBS and parathyroid hormone (PTH) levels. Signaling through the PTH1R has been suggested as one of the main pathways that regulate Dkk-1 in AS patients and other inflammatory conditions (32).

In our opinion, it should be emphasized that the contradictory data on Dkk-1 in AS could be, at least in part, related to the variability of PTH and of vitamin D, one of its main determinants, whose metabolism could be impaired in inflammatory conditions (33).

Another relevant category of hormones implicated in rheumatic condition are sex hormons and nowadays gender medicine is an increasing topical issue.

The male-to-female ratio of SpA ranges approximately from 1:1 in patients with non radiographic-axSpA and 2:1 in patients with AS (34) and has been demonstrated that the proportion of female patients is significantly lower among the patients who progressed from nr-axSpA to AS (35). Furthermore male patients with AS have more severe radiographic damage than female (36).

Even if underlying biological differences between men and women with AS are still unknowns, estrogens are known to modulate T cell differentiation, type 2 cytokine production (37) and, in animal model, the inhibition of the differentiation of T helper17 cells (38).

Gooren et al. reported in 22 male AS patients reduced levels of testicular testosterone reserve, elevated levels of LH, inversion of estradiol testosterone ratio and slightly increased estradiol. Levels of estrogens (17β-estradiol) have been founded lower in active AS patients disease than in those in remission state (39). To explore this latter evidence, Jeong and colleagues have demonstrated how estrogens can suppress the development of arthritis in SpA mouse model, probably because estrogens inhibit Wnt signaling (40).

## Rheumatoid Arthritis

RA is a chronic inflammatory disorder that leads to severe joint damage and disability (41). The most studied type of bone involvement in RA is the focal cortical bone loss (erosions). However, we can indeed label RA as a “bone disease”: cortical erosions, systemic bone loss (osteoporosis), and periarticular bone loss contribute to the disease burden (**Figure 1**). Local and systemic inflammation suppresses both bone formation and erosion healing (42, 43). Focal bone impairment is the result of the interaction between the dysregulated inflammation of the synovial membrane (synovitis) with the surrounding bone microenvironment. Eventually, this induces an excessive differentiation and activation of the osteoclast line and the development of cortical erosions (44).

At the systemic level, pro-inflammatory cytokines such as TNF-alfa, interleukin1(IL-1), and interleukin 6 (IL-6) play a crucial role in systemic bone impairment throughout, mainly *via* the RANK/RANKL/OPG system (45).

Among the hormones involved in the pathogenesis of RA, data suggest that PTH could play a significant role in bone erosions.

Indeed, in RA patients has been documented an association between joint erosions and higher PTH levels (10), probably in a vitamin D-independent way. T lymphocytes are hypothesized to promote PTH-induced osteoclastogenesis by increasing the medullary stromal cell responsiveness to PTH itself (46). As shown in both healthy and in subjects with primary hyperparathyroidism (47), persistent PTH signaling increases the RANKL/RANK pathway activity (48), negatively correlates with sclerostin and positively with Dkk-1.

As seen in AS (49), in mastocytosis with bone involvement (50), and in other conditions, also in RA PTH is suspected to be one of the main determinants of Dkk-1 serum levels (32). Furthermore, in RA patients, Dkk-1 and PTH serum levels are significantly higher, despite therapy with glucocorticoid (GC), tumor necrosis factor-alfa inhibitors (TNFi) or bisphosphonates (BPs) (32).

Different studies have remarked that levels of Dkk-1 in RA patients correlate inversely with BMD, in particular at cortical bone sites (32) and a significant association between low BMD and focal bony erosions has been described (51, 52). RA patients are generally characterized by increased diffuse bone loss together with a higher risk for hip and vertebral fractures (53).

Simon and colleagues analyzed in patients affected by RA the quantity and quality of intra-articular bone of the metacarpal heads (54), which are one of the typical erosion sites (55). In their study, a significant trabecular and cortical intra‐articular bone loss was observed, similar to the impairment that characterizes the bone microstructure after the menopause (54). These microstructural features in RA patients correlate with disease activity, with serum levels of pro‐inflammatory cytokines and, as already mentioned, with serum levels of Dkk-1 and PTH, as a consequence of a common pathological mechanism of both inflammatory and metabolic nature (32, 56, 57). For these reasons, we speculated that osteoporosis might be a significant and independent determinant of bone erosions in RA (10). Similar *intra*-articular microstructural alterations can also be secondary to aging and/or estrogen deficiency, and have also been found in *extra*-articular bone, such as the radius (58). Moreover, erosions and low mineral density share many risk factors: anti-citrullinated protein antibodies (ACPAs) (59, 60), disease activity, cigarette smoking, alcohol consumption, hypovitaminosis D, corticosteroid use, and aging (44). In line with this hypothesis, some osteoporosis treatments seem to prevent erosions in RA. Denosumab (Dmab), a monoclonal antibody that blocks RANKL, has been demonstrated to stop the structural progression in RA (61). Furthermore, it determines the increase in bone mass, especially in trabecular bone areas, regardless of GC use (62).

The keystone that allows Dmab to perform this protective effect is the inhibition of the osteoclast, a multinucleated cell that contributes not only to the development of erosions in the subchondral bone tissue but also to the destruction of the mineralized cartilage (61).

In our opinion, overlooking the bone metabolic status in osteoporotic RA patients might be one of the key determinants in those patients whose erosions continue to progress despite adequate treatment with DMARDs (63). To date, the meta-analyses on effects of DMARDs on radiographic progression of RA patients lack of BMD data, and this might represent an important bias (63).

Teriparatide (TPTD), a PTH analog, is an effective drug used in severe osteoporosis, with a completely different action than Dmab (64). TPTD boosts bone formation biomarkers already from the first month of treatment (64). Interestingly, long-term stimulation with PTH analogue seems to increase serum Dkk-1 in women postmenopausal osteoporosis (65). And this observation is consistent with the demonstrated relationship between serum levels of PTH, serum levels of Dkk1 and bone erosions in RA patients (32). These data suggest that PTH might contribute to determinate local DKK1 over-expression. Hence, high serum PTH levels may enhance local bone resorption and hinder bone repair by promoting DKK1 expression (32). Besides the prevention of erosions, there is still controversy on the possibility of their repairment over time. Apparently, this might occur in some cases (66).

In different settings, TPTD has been suggested as a possible treatment to enhance fracture healing. Furthermore, in a TNF-transgenic murine model of RA, combination therapy with TPTD and TNFi was associated with erosions repair (67). Unfortunately, in a recent trial on RA subjects, this endpoint was not achieved (68, 69). To explain this finding, one should remember that the bone microenvironment might be permanently altered in the setting of a longstanding inflammation. Therefore, this might cause a permanent impairment in the number and differentiation potential of the osteoblast progenitors (70), on which TPTD is supposed to exert its function. Furthermore, the chronically high serum PTH levels are associated with increased bone porosity (71) and decreased cortical thickness, contributing to the explanation for these disappointing results (68).

Ebina and colleagues compared the effects on joint erosions of the three different classes of therapies (BPs, Dmab, and TPDT) in biologic-naïve RA patients. Switching from BPs to Dmab was found to be more effective than continuing BPs or switching to TPTD in the prevention of structural progression (72).

As already mentioned, pro-inflammatory cytokines activate the osteoclast line *via* the RANK/RANKL/OPG axis, and are directly involved in bone complications (45). Pro-inflammatory cytokines are currently the main targets of the most widely used drugs for RA, i.e. TNFi and anti-IL6 receptor (IL-6R) monoclonal antibodies.

TNFα contributes substantially to RA pathogenesis, and it is involved in many pathways (73). Interestingly, some studies have demonstrated its relationship with bone turnover cytokines (9, 74, 75). TNF-alfa increases Dkk-1 levels in synovial fibroblasts both *in vitro* and *in vivo*, and it is correlated with the presence (9, 74) and the progression (75) of bone erosions.

On the other hand, in RA patients, TNFα inhibition demonstrated bone metabolic effects by reducing Dkk-1 and sclerostin serum levels (76, 77).

In RA, also IL-6 is strongly involved in bone loss through the inhibition of the Wnt canonical pathway (78). A recent study investigated the short-term effects of an anti-IL-6 treatment (tocilizumab) on bone turnover markers (BMTs) in RA patients, comparing it with TNFi and GCs (methyl-prednisone) (79). The strong and prompt influence of TNFis on bone turnover markers seen in previous studies (76) was not observed with the IL-6R blockade. Indeed, no significant change was observed either for sclerostin or Dkk-1 in the tocilizumab arm, while the arm receiving TNFis showed a decrease in markers of bone resorption and an increase in the markers of bone formation (80). To explain this difference, the Authors hypothesized a slower influence of tocilizumab on the Wnt pathway.

As seen in other arthritis, sex hormones, especially estrogens, are closely involved in RA. RA is more prevalent in women with a female-to-male sex ratio of 4:1 (81), even if the reason is still partially unclear. Estrogens can have both stimulatory and inhibitory effects on the immune system, as described by Straub (82), and estrogen exposure has been associated with increased risk of RA due to pro-inflammatory action of these hormones unbalanced with the anti-inflammatory one of androgens (82). However it must be noted that two conditions characterized by a low level of estrogens such as menopause and the use of anti-estrogen agents have been associated with an increased risk of developing RA (respectively HR, 2.1; (95% CI, 1.5–3.1) and OR, 2.4 (95% CI, 1.9–3.0) and OR, 1.9 (95% CI, 1.6–2.1) depending by dose and time of anti-estrogens exposure) (83–85). Furthermore, long duration of pharmacological estrogen exposure under oral contraceptives (OCs), seems to protect from the development of the disease, with a cumulative positive dose effect (86–88). Also the pregnancy condition has been described as protective against RA, probably thanks to a balance between progesterone to the high level of estrogens (89–91). On the contrary the post-partum and the lactating period, characterized respectively by a decline of estrogens and by the release of prolactin (PRL), has been consistently associated with an increased risk of RA (92). Captivatingly, in a small study, levels of 17β-estradiol and other hormones (progesterone, aldosterone and growth hormone) were founded higher in synovial fluid (SF) of RA patient in comparison to patients with osteoarthritis (93), suggesting a influencing roles also at local sites.

PRL is a sex hormones, mainly secreted by pituitary gland and with pleiotropic functions, among which the capacity to enhance or inhibit pro-inflammatory cytokine production (94). PRL can be locally produced by macrophages, T cells and synovial fibroblasts and its receptor (PRLR) is expressed in synovial macrophages and lymphocytes (94). For those reasons, PRL have long been thought to play an important role in RA disease (95, 96) even if there are contradictory results about its serum levels, that have been founded raised in RA patients compared to healthy controls in some studies (97–100). Levels of PRL has been dosed also in SF of RA patients without finding significant differences with patients with osteoarthritis (93). Nevertheless PRL and its receptors were suggested by Tang and colleagues to be engaged in RA thanks to a local crosstalk, *via* auto- or paracrine ways, between the immune and endocrine systems. The local presence of PRL system in synovial tissue of RA (and PsA, as we will see later) patients has been propose as a new potential therapeutic target (94).

## Psoriatic Arthritis

PsA is a chronic, systemic inflammatory disease that affects peripheral joints, the axial skeleton, and it is associated with psoriasis of the skin and nails (101).

The bone involvement in PsA patients differs from the one seen in RA, presenting erosive damage associated with exuberant bone formation, especially in entheseal sites (102) (**Figure 1**). The different bone involvement in RA and PsA may be explained by a diverse interplay of the involved mediators and cytokines.

PsA is a strongly IL-17-driven disease (103). Among its functions, IL-17 is a potent osteoclastogenetic factor (104), particularly at inflamed sites undergoing mechanical stress, such as the entheses.

Concerning the etiopathogenesis of enthesopathy, it is well known that local trauma and inflammation play a pivotal role in the T cells activation, especially the gamma-delta subset (105). Cytokines such as IL-23 and IL-17 stimulate resident cells (chondrocytes, osteoblasts, and, to a lesser extent, osteoclasts) to secrete metalloproteinase and to overexpress the RANK-RANK-ligand, leading to both erosion and bone formation (106). However, entheses are poor in osteoclasts, and this consideration might suggest one explanation for a focal unbalance between bone formation and erosions. Furthermore, the mechanical stress in the synovial-entheseal complex might contribute to promote bone formation (19).

The relationship between anti IL-17 therapy and bone metabolism in PsA has been studied in a small longitudinal study in which Dkk-1 and sclerostin levels increased after treatment with secukinumab in a cohort of PsA patients, suggesting a possible drug-induced inhibition of local bone over-proliferation (107).

Conversely, one meta-analysis reported benefits in terms of BMD under TNFi treatment, thanks to the suppression of systemic inflammation (108). Over the past years, the relationship between inflammation and the consequent role of TNFi in the structural progression has been greatly debated both in AS (109) and PsA (110).

According to older RCTs, TNFi treatments failed to control the radiographic progression, despite the achievement of clinical improvement (111). For this reason, a few years ago, the so-called “TNF brake” hypothesis was proposed: early in the pathogenesis, TNFα might upregulate Dkk-1 expression. However, when the inflammatory lesions are established and mature, the bone microenvironment undergoes some other changes, activating bone formation pathways with the consequent expression of bone growth factors.

In this setting, TNFα has been hypothesized to act as a brake slowing down the new bone formation through the Dkk-1 upregulation. This model was postulated to explain why the TNFi failed to prevent radiological progression (i.e. syndesmophytes) (109). Nowadays, some new data suggest the efficacy of TNFis on radiological damage if started in a timely fashion. The suppression of local inflammation from the very beginning is thought to prevent the activation of bone formation pathways (112, 113).

Finally, in all inflammatory arthritis, the occurrence of physiological aging and senile osteoporosis can alter the function of bone mechanoreceptors, whose dysfunction can contribute to bone impairment through the impairment of different pathways that eventually converge on Wnt signaling (114).

As seen for RA, PRL has been studied also in PsA. As mention above for RA, PRL is locally expressed in the synovial tissue also of PsA patients and PRL mRNA expression positively correlates with disease activity (94).

The PRL-PRLR binding activates various signaling pathways among which the Janus kinase/signal transducer and activator of transcription (JAK-STAT) one, once the more recently studied therapeutic target both for RA and PSA.

Not many studies have faced the role of sex hormones in PsA, a disease with sex ratio of 1:1 and a bimodal distribution in female sex, with peaks of incidence during late adolescence and the perimenopausal period (115). Literature shows how psoriasis often improves during pregnancy and reappear in the post-partum, suggesting a direct link between estrogen and progesterone and disease severity (115). but less is known about PsA.

## Other Rheumatic Conditions

The strong link between hormones, bone turnover, and rheumatic diseases has been described not only in the chronic arthritides but also in other rheumatic conditions, such as Polymyalgia rheumatica (PMR), Crystal-Induced Arthritides, and even connective tissue diseases (i.e. Systemic Erythematous Lupus, Sjogren Syndrome and Systemic Sclerosis) (116–119).

PMR is a chronic inflammatory disease affecting older adults that causes pain, stiffness, and inflammation of the shoulder and pelvic girdles, mainly treated with GCs (120). Data on the changes of bone metabolism induced by GCs and on the profile of bone markers its fine regulators (i.e. Dkk-1, sclerostin) in PMR are scarce.

In a very recent study (116), we showed an increase in Dkk-1 serum levels in also treatment-naive PMR patients. In this study, we also observed a significant decrease of Dkk-1, together with C-terminal telopeptide of type-1 collagen (CTX, a bone resorption marker) and in N-propeptide of type I collagen (PINP), after one month of GCs therapy. A similar trend was documented for sclerostin.

Systemic sclerosis (SSc) is a connective tissue disease characterized by tissue fibrosis and microvascular involvement. Even if difficult to assess due to the heterogeneity of the cohorts, it seems that SSc patients have an increased risk of developing osteoporosis (121). The most interesting aspect of bone involvement is that the modulators of bone metabolism are partially involved in SSc pathogenesis. The Wnt system plays a significant role in the development of fibrosis (122), through the endothelial-to-mesenchymal transition (117). For this reason, data about bone turnover markers are often conflicting, especially on Dkk-1. For instance, one study found a correlation between elevated Dkk-1 and low TBS (123), while in another, a similar correlation has been found with modified Rodnan Skin Score (mRSS) but not with BMD (124).

Furthermore, in SSc patients, increased serum RANKL levels have been observed (125), and TRIAL, a ligand of OPG with vascular protection properties (126), was found to be higher in SSc compared to the general population, suggesting a possible link between microvascular damage and bone loss.

Systemic Lupus Erythematosus (SLE) is an autoimmune connective tissue disease with a complex pathophysiology and a spectrum of clinical manifestations involving potentially every organ and system of the body (127).

As seen above in RA, bone tissue could be affected both at the systemic level, with low BMD and fragility fractures, and at the focal site, with joint erosions (118). Furthermore, the bone loss could be secondary to the disease itself and/or to steroid treatment (128), and its pathophysiology is extremely heterogeneous: its takes into account systemic inflammation, impairment of vitamin D-PTH-calcium system both for limited sun exposure and altered renal function, impairment of sex hormones (i.e. dehydroandrostenedione) (129). A two to threefold increased fracture risk has been reported in several large cohorts (130).

Decreased osteocalcin serum levels (a marker of bone formation) and increased levels of CTX in untreated premenopausal SLE patients have been observed, with a correlation between osteocalcin and disease activity (119).

A dysregulation in the Wnt/beta-catenin signaling has been observed in B and T cells involved in SLE pathogenesis and among bone mediators, Dkk-1 has been suggested as a potential biomarker for bone erosions (118) and as an independent biomarker for lupus nephritis (131).

## Conclusions

Over the last years, rheumatology, endocrinology and immunology have intertwined especially with the domain of bone metabolism. This new field of research is providing new data that are contributing to the development of the evolving pathophysiological models of the rheumatic diseases. RA could be labled as “bone disease” in which have been described the connection between pro‐inflammatory cytokines, PTH, Dkk-1, bone erosions and bone loss. Some osteoporotic treatment, i.e. denosumab, demonstrates to stop the structural progression (61). In PsA, IL-23 and IL-17 interact with bone system through chondrocytes, osteoblasts, and osteoclasts and *via* RANKL/RANK signaling, leading to both erosion and bone formation. And in AS, the Wnt pathway seems to be one of the key players involved in the relationship with bone metabolism, characterized by pathological neoformation and impaired bone mineral density (20, 21). Hopefully, a deeper understanding of the relationship among bone turnover, hormones and the different rheumatic conditions’ phenotypes will be able to improve the clinical and therapeutic management of our patients.

## Transparency declaration

The lead author (the manuscript’s guarantor) affirms that the manuscript is an honest, accurate, and transparent account of the study being reported; that no important aspects of the study have been omitted; and that any discrepancies from the study as planned (and, if relevant, registered) have been explained.

## Author Contributions

EB conceived and wrote the article. MR, GO, OV, DG, and AF revised the article critically for important intellectual content. All authors contributed to the article and approved the submitted version.

## Funding

This research received no external funding.

## Conflict of Interest

The authors declare that the research was conducted in the absence of any commercial or financial relationships that could be construed as a potential conflict of interest.
